# Endoscopic cartilage tympanoplasty: full thickness and partial thickness tragal graft^☆^^☆☆^

**DOI:** 10.1016/j.bjorl.2018.12.006

**Published:** 2019-02-20

**Authors:** Kartik Parelkar, Vandana Thorawade, Hetal Marfatia, Devika Shere

**Affiliations:** a Grant Government Medical College & Sir J.J. Hospitals, Mumbai, India; b K.E.M. Hospital, Department of ENT, Parel, Mumbai, India; c Rajiv Gandhi Medical College & Chhatrapati Shivaji Maharaj Hospital, Kalwa, Thane, India

**Keywords:** Cartilage, Endoscopic, Tympanoplasty, Perforation, Tympanic membrane, Cartilagem, Endoscópico, Timpanoplastia, Perfuração, Membrana timpânica

## Abstract

**Introduction:**

Cartilage is the grafting material of choice for certain disorders of the middle ear. The indications for its routine use remain controversial due to the possible detrimental effect on post-operative hearing.

**Objective:**

The present study was carried out to report a personal experience with “tragal cartilage shield” tympanoplasty to compare the results, in terms of graft uptake and hearing improvement, of endoscopic cartilage shield technique using either partial thickness or full thickness tragal cartilage for type 1 tympanoplasty and to highlight the tips for single-handed endoscopic ear surgery.

**Methods:**

Fifty patients with safe chronic suppurative otitis media, assisted at out-patient department from February 2014 to September 2015 were selected. They were randomly allocated into two groups, 25 patients were included in group A where a full thickness tragal cartilage was used and 25 patients included in group B where a partial thickness tragal cartilage was used. Audiometry was performed 2 months after the surgery in all cases and the patients were followed for one year.

**Results:**

Out of the total of 50 patients 39 (78%) had a successful graft take up, amongst these 22 belonged to group A and 17 belonged to the group B. The hearing improvement was similar in both groups.

**Conclusion:**

This study reveals that endoscopic tragal cartilage shield tympanoplasty is a reliable technique; with a high degree of graft take and good hearing results, irrespective of the thickness. Furthermore, the tragal cartilage is easily accessible, adaptable, resistant to resorption and single-handed endoscopic ear surgery is minimally invasive, sutureless and provides a panoramic view of the middle ear.

## Resumo

**Introdução:**

A cartilagem é o material de enxerto de escolha no tratamento cirúrgico de certas condições clínicas da orelha média. Devido ao possível efeito prejudicial na audição pós-operatória, as indicações para seu uso rotineiro ainda são controversas.

**Objetivo:**

Relatar a experiência dos autores com a timpanoplastia tipo 1 endoscópica usando cartilagem tragal e comparar os resultados entre a cartilagem tragal com espessura parcial e espessura total, em termos de integração do enxerto e melhoria da audição. O estudo também buscou apresentar sugestões para cirurgia endoscópica de orelha média com uma única mão (*single-handed endoscopic ear surgery*).

**Método:**

Foram selecionados 50 pacientes com otite média crônica supurativa, atendidos neste ambulatório entre fevereiro de 2014 e setembro de 2015, alocados aleatoriamente em dois grupos: 25 pacientes foram incluídos no grupo A, no qual uma cartilagem tragal de espessura total foi usada e outros 25 pacientes foram incluídos no grupo B, no qual foi usada uma cartilagem tragal de espessura parcial. Em todos os casos, uma audiometria foi feita dois meses após a cirurgia; os pacientes foram acompanhados por um ano.

**Resultados:**

Dos 50 pacientes, o enxerto foi bem-sucedido em 39 (78%), entre os quais 22 pertenciam ao grupo A e 17 pertenciam ao grupo B. A melhoria da audição em ambos os grupos foi muito semelhante.

**Conclusão:**

O estudo indicou que a timpanoplastia endoscópica com cartilagem tragal é uma técnica confiável, com alto grau de integração do enxerto e bons resultados de audição, independentemente da espessura usada. Além disso, a cartilagem tragal é facilmente acessível, adaptável e resistente à reabsorção; a cirurgia endoscópica é minimamente invasiva, sem sutura e proporciona uma visão panorâmica da orelha média.

## Introduction

The tragal cartilage is often preferred for revision surgeries, attic reconstructions, atelectasis and cases of suspected eustachian tube dysfunction. The use of tragal cartilage in middle ear surgery is not a new concept however, during the last decade it has gained greater approval across the globe. The main reason behind this is not only its reliability and stability but also the growing demand for minimally invasive procedures. The tragal cartilage is an excellent graft especially in endoscopic tympanoplasties. The transcanal approach is scarless and the field is bloodless. The cartilage shield being relatively rigid is possible to place single handedly with ease and precision. The single main controversy that the use of this graft faces is related to its thickness. The literature on the acoustic benefits of slicing the tragal cartilage is scant. Technique of SHEES, advantages and disadvantages of slicing the cartilage have been discussed in this article.

## Methods

Patient population and evaluation

From February 2014 to September 2015, endoscopic tragal cartilage tympanoplasty was performed in 50 patients (21 females and 29 males, age range 18–70 years) who presented to the ENT out-patient department of our tertiary-care hospital.

Patients with safe type of Chronic Suppurative Otitis Media (CSOM) and conductive hearing loss were chosen. Those with unsafe disease, active infection, sensorineural hearing loss, recurrent perforations and co-morbidities like diabetes or compromised immune system were excluded.

These cases were randomly allocated into Group A and Group B of 25 patients each. Patients in Group A underwent endoscopic Type 1 tragal cartilage tympanoplasty with a full thickness graft (∼0.9 mm) while those in the Group B underwent the same procedure with a partial thickness graft (∼0.4 mm). All the cases had a dry ear for atleast 2 months pre-operatively and an intact ossicular chain.

The following parameters were assessed after 2 months of surgery: graft take-up rate and the hearing improvement in both the groups. After this assessment the patients followed up for routine ear examination up to 1 year. A successful result was defined as having closure of perforation, no medialization or lateralization and having good vascularity over the cartilage graft. Failure included residual/recurrent perforation and no hearing improvement or deterioration in hearing. The Pure Tone Audiogram (PTA) testing calculated the average hearing at 500, 1000 and 2000 Hz pre-operatively and 2 months post-operatively in all cases. Institutional ethical review board permission (UIN: 3256) and patients consent were obtained for this study.

### Surgical procedure

**Figure 1:**
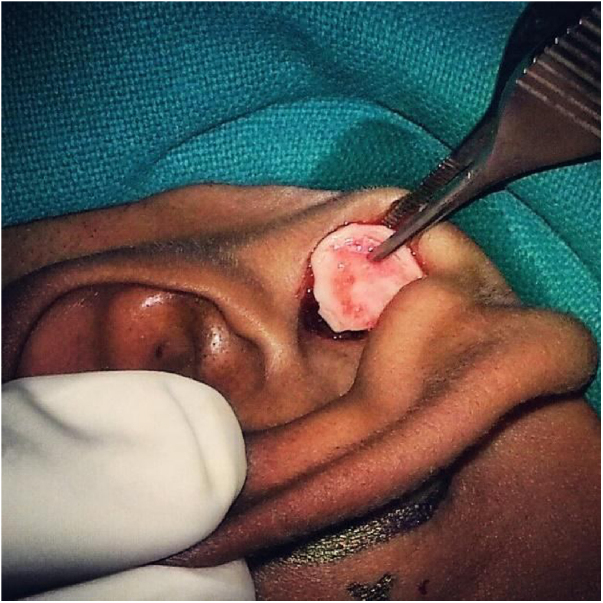
A full thickness tragal cartilage with perichondrium on both sides being harvested.

All the patients were meticulously investigated for anaesthesia fitness and a routine diagnostic nasal endoscopy was performed. The middle ear was examined with a 4 mm zero degree endoscope and a PTA were done pre-operatively. The tympanoplasties were done under local anaesthesia using infiltration with 2% lignocaine and adrenaline supplemented by sedation when needed. An inverted U-shaped incision was made on the skin over the tragus leaving a 2 mm rim at the dome of the cartilage. The inverted U-shaped skin flap was then elevated and a tragal cartilage graft was harvested with perichondrium on both sides (Fig. 1). The bed from where the graft was harvested was mopped with plain adrenaline soaked merocel piece and the skin flap reposited.

**Figure 2:**
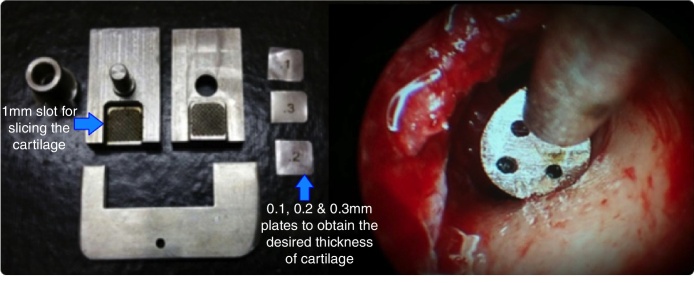
Cartilage slicer used for slicing the tragal cartilage uniformly and a round knife with sieve and an inbuilt suction for SHEES.

Using a 4 mm zero degree endoscope the perforation was visualized and the margins were freshened to promote good capillary flow. A micro-sickle knife was used for this purpose. Haemostasis and a good anaesthetic effect were achieved using 4% lignocaine and plain adrenaline soaked merocel pieces. The graft was shaped and sized according to the case and a small v-shaped notch was made at 12 o’clock. The cartilage with full thickness and perichondrium on both sides was used for the patients in Group A while the graft was sliced to a partial thickness (∼0.4 mm) for the patients in Group B. The sliced cartilage had perichondrium only on one side and curled towards the same side. A cartilage slicer was used to slice the graft uniformly and to know its exact thickness (Fig. 2).

A Rosen's incision was taken and the Tympanomeatal (TM) flap was raised using a round knife with sieve and suction specifically used for SHEES (Fig. 2). Once the annulus was elevated the ossicular mobility was checked and gelfoam was kept in the region of the sinus tympani and the eustachian tube opening.

The fashioned graft was then placed over the handle of malleus and under the annulus in an underlay manner. In Group B, the sliced cartilage graft was placed such that the side with intact perichondrium was lateral. The TM flap was then reposited and the ear canal packed with gel foam and a piece of merocel. The reposited skin over the tragus was not sutured. A small cotton dressing was given externally.

### Post-operative care

Patients were advised to take water precautions and avoid vigorous nose blowing. The external cotton and merocel in the ear canal was removed after 1 week and antibiotic-steroid ear drops were started then. Patients were kept on oral antibiotics for 1 week. The gelfoam was allowed to resolve gradually on its own. The operated patients were examined at 1 week, 3 weeks and then 2 months post-operatively. A PTA was repeated at 2 months once a viable graft was visible and patient was asymptomatic. These patients followed up for routine ear examination up to 1 year after the surgery.

Patients who had upper respiratory tract infection and otitis media with effusion post-operatively were managed with nasal steroidal sprays and an effort was made to prevent these conditions from hampering the take of the graft.

## Results

### Graft take-up

**Figure 3:**
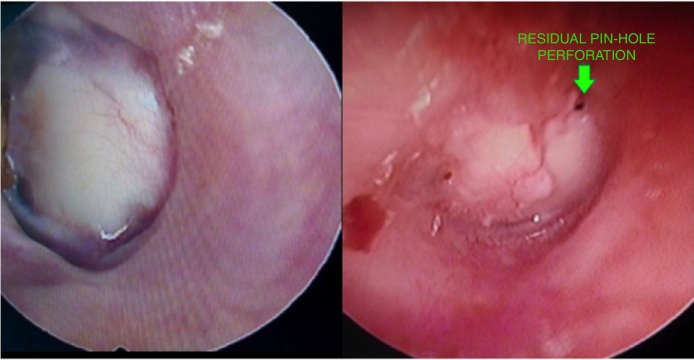
A successful full thickness tragal graft take-up and pin-hole residual perforations at the edge of a partial thickness tragal graft.

Out of the total of 50 patients 39 (78%) had a successful graft take up (Fig. 3), amongst these 22 (88%) belonged to the full thickness i.e. Group A and 17 (68%) belonged to the partial thickness i.e. Group B. Among the 11 cases which had problems with respect to the take of the graft, 3 (12%) belonged to Group A and 8 (32%) belonged to Group B. Of these, all 3 of the Group A and 3 of the Group B had small pin-hole residual perforations at the edge of the cartilage graft (Fig. 3) while remaining 5 of the Group B had either medialization of the graft or a large residual. The graft take up rate of full thickness cartilage was significantly better than that of the partial thickness graft; *p*-value = 0.017 (Table 1). This take up rate essentially remained the same at 1 year follow-up too.

**Table 1:** Comparison of graft uptake between full thickness graft (A) and partial thickness graft (B).

Take up	Group		Total
	A	B	
Fail	3	8	11
	12.0%	32.0%	22.0%

Good	22	17	39
	88.0%	68.0%	78.0%

Total	25	25	50
	100.0%	100.0%	100.0%

*p*-value = −0.017 (Sig.).

Fisher exact test was used for calculation.

The table shows the comparison of graft take rate in both the groups. The uptake rate of full thickness was 88% and that of partial thickness was 68% which is statistically significant; the take-up with full thickness graft is better than that with partial thickness graft.

### Acoustic benefit

**Figure 4:**
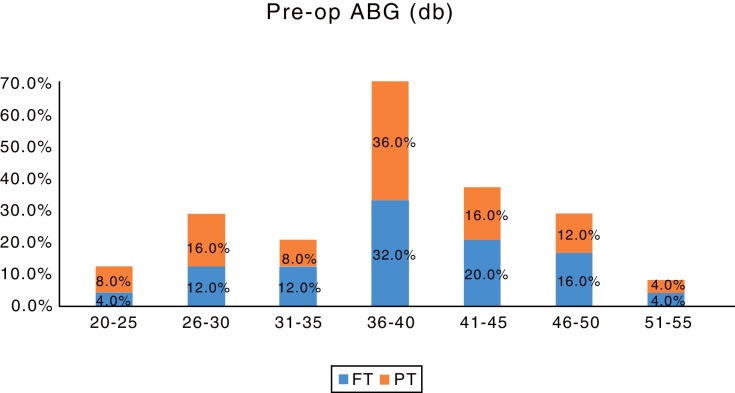
Bar chart showing the pre-operative air-bone gaps in both the groups. FT, Full thickness; PT,Partial thickness tragal cartilage tympanoplasty.

**Figure 5:**
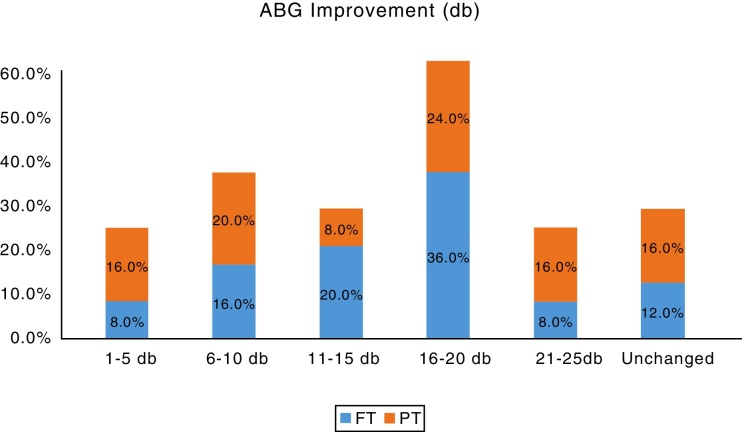
Bar chart showing the improvement in the air-bone gap post-operatively in both the groups. FT, Full thickness; PT,Partial thickness tragal cartilage tympanoplasty.

The average pre-operative hearing was 40.80 ± 7.46 dB for Group A and 39.40 ± 7.95 dB for Group B. The post-operative PTA done 2 months after surgery showed an average hearing of 26.72 ± 8.08 for Group A and 26.40 ± 8.60 for Group B. The hearing improvement in both the groups was very similar and statistically significant compared to the pre-operative hearing (*p*-value = 0.012 for Group A and *p*-value = 0.018 for Group B) (Table 2). The details hearing status in terms of Air-Bone Gap (ABG) are shown in (Figure 4, Figure 5). The PTA was not repeated at 1 year follow up, as these patients did not note any significant change in their hearing levels, compared to the hearing assessment done 2 months after the surgery.

**Table 2:** Pre-op hearing and post-op hearing in full thickness graft and partial thickness graft.

Hearing (db)	Group	*N*	Mean	STANDARD DEVIATION	*p*-value
Group A	Pre-op	25	40.80	7.46	0.012 (Sig.)
	Post-op	25	26.72	8.08	

Group B	Pre-op	25	39.40	7.95	0.018 (Sig.)
	Post-op	25	26.40	8.60	

Mann–Whitney *U* test was used for calculation.

The table shows that the hearing improvement in both the groups was very similar and statistically significant compared to the pre-operative hearing.

## Discussion

### About the tragal cartilage

The use of cartilage is experiencing a renaissance in ear surgery because of its reliability as a graft material and also the increasing use of endoscope in ear surgeries. The tragal cartilage is nourished by diffusion and eventually becomes incorporated in the tympanic membrane.^1^ The graft is easily accessible, adequate and its rigidity makes it easy to fashion and manipulate, reducing the learning curve in endoscopic tympanoplasty. In our patients we have used an inverted U-shaped incision to raise a skin flap over the tragus. This technique, unlike the routine longitudinal incision used to harvest the graft, allows us to harvest adequately sized, circular shaped cartilage with more precision.

### Tips for SHEES

The 4 mm zero degree endoscope also allows a panoramic view of the three main elements in tympanoplasty surgery: The ear canal, tympanic membrane, and the tympanic ring.^2^

All the 50 cases in our study were operated using a 4 mm zero degree nasal endoscope. In our experience, the shorter otoendoscopes have a disadvantage, as there is crowding of hands, as both the hands are in the same horizontal plane and the lesser diameter compromises the view. The 4 mm zero degree endoscope can be used for endoscopic nasal surgeries and SHEES too, hence is cost-effective and has a monetary benefit. A bloodless field is essential in SHEES. We have used merocel pieces while raising the TM flap instead of cotton pledgets. This prevents cotton fibres from remaining back in the middle ear or the ear canal and affecting the result of the surgery. We have also used merocel to pack the external part of the ear canal and keep the tragal skin flap in place once the surgery was complete. An exact count of the merocel pieces must be kept as they cannot be left behind in the surgical field. Fogging of the endoscope compromises the vision; this can be reduced by placing a small piece of gelfoam at the eustachian tube opening, before starting the surgery itself. Frequently dipping the scope tip in savlon helps in defogging too and reduces the heat generated by the endoscope, especially when a xenon light source is being used.^2^

### Instrumentation

There are certain specific endoscopic instruments that we have used in our patients like the micro-sickle and the round-knife with sieve and an integrated suction (Fig. 2). The micro-sickle and micro-flag knife allows us to freshen the margins of the perforation and lift the annulus meticulously, even in cases with a narrow canal and anterior canal wall overhang. These instruments do not obstruct the endoscopic view and are not expensive too. The round-knife with sieve and suction was used while raising the TM flap. Though the suction tube attached to this instrument caused some discomfort while using it initially, over a certain period of time the surgeons got accustomed to it. There are many variations of this instrument available in the market but in our opinion one must select the perfect one, which should have a minimal space and angulation between the round-knife and the suction tip.

There are certain other techniques to manage patients with a narrow ear canal like the Tarabichi's stitch, which straightens the ear canal and increases the working space with the endoscope. One can also use an endoscope holder if the use of two hands is essential to the surgery. However in our experience, simple cartilage tympanoplasties can be efficiently managed by using the above mentioned instruments by the single handed technique itself.

### Full thickness vs. partial thickness graft

The graft take up rate is not an issue with tragal cartilage, the only controversy this material faces is its effect on the hearing owing to its thickness. Zahnert et al. examined the frequency response function of the tragal cartilage plates using a laser Doppler interferometer. There were transmission losses at lower frequencies when large tympanic membrane defects were reconstructed with thick pieces of cartilage. They concluded that a cartilage plate with a thickness of less than 0.5 mm gave the least acoustic transfer loss.^3^

Atef et al. performed a prospective clinical study using full thickness and partial thickness cartilage-perichondrium composite island grafts in patients with central perforation and intact ossicular chain, who were allocated randomly into two groups. In the first group 30 patients were treated with full thickness grafts, with one recurrent perforation. In the second group half thickness grafts were used in 32 patients with one recurrent perforation. The anatomical results were good with failure of 3% in both groups. The post-operative hearing was same in both groups.^4^

Vadiya and Bhatt stated that the graft take up rates are excellent for both partial and full thickness tragal cartilage in modified cartilage shield technique of tympanoplasty. Difference in hearing gain is not statistically significant between the two groups, except at 4000 Hz where hearing gain in partial thickness tragal cartilage recipients is more than full thickness tragal cartilage recipients.^5^

Our study contradicts the work of Zahnert et al. and is in agreement with that of Atef et al., demonstrating similar hearing improvement irrespective of the thickness of the tragal cartilage graft.

**Figure 6:**
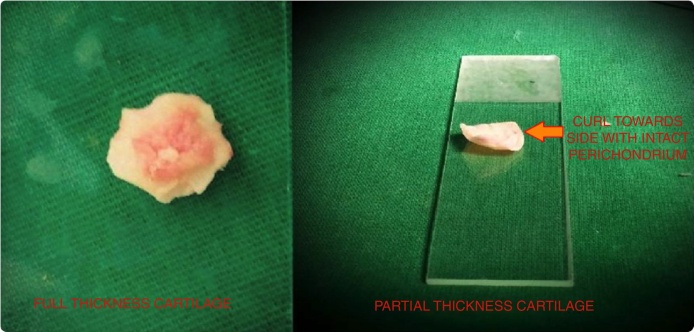
The full thickness graft with no curl and a partial thickness tragal cartilage graft curling towards the side of the attached perichondrium.

We attribute the higher rate of pin-hole residuals at the edges of the sliced cartilage graft in patients belonging to Group B, to the major issue of “curling”. On slicing the cartilage graft the perichondrium contracts and causes the edges of the graft to curl to the same side (Fig. 6). This curled graft is difficult to place in an underlay manner compared to the full thickness graft according to us. There is hardly any literature which addresses the curling problem, Tos has mentioned four incisions of the perichondrium – “the anti-curling incisions” which may help to solve this.^6^ If the graft is placed with perichondrium on the medial surface, there should be no trauma to the mucosa over the promontory to avoid middle ear adhesions. We preferred to place the sliced cartilage grafts with intact perichondrium on the lateral side in our cases belonging to Group B.

### Limitation of our study

There have been many studies reporting 100 percent or very high take up rates in primary tympanoplasties with tragal cartilage as the graft of choice. In our study the overall take up rate is 78%, we attribute this lower rate not only to patient factors such as low socio-economic class, that our hospital caters but also to the learning curve of this techniques. Being a teaching tertiary-care hospital, not all surgeries were done by a single senior surgeon; this also could have been one of the factors in our opinion, though the technique and protocol of the procedure essentially remained the same. In order to reduce the learning curve for endoscopic ear surgery, we encourage our junior resident doctors to routinely use the 4 mm zero degree endoscope for diagnostic examinations of the ear. Once they develop a good hand-eye co-ordination they are trained to go-ahead with minor procedures.

A potential drawback of the tragal graft is its opacity, as it may be more difficult to detect an iatrogenic cholesteatoma or post-operative secretory otitis media. We have had two of our failures due to secretory otitis media too. These patients had evidence of glue coming out from the residual perforation at the edge of the cartilage graft. However, the temporalis fascia too is often not completely transparent post-operatively.

## Conclusion

Single Handed Endoscopic Ear Surgery (SHEES) with tragal cartilage graft is one of the best modality to manage patients who require a simple Type 1 tympanoplasty. It is minimally invasive, scarless, time-saving, cost-effective and has a high patient compliance in our opinion.

The thickness of the tragal cartilage does not affect the hearing results significantly according to our results. The condition of the middle ear, patient selection and the technique of the surgery along with post-operative care are responsible for a successful result rather than the graft thickness.

## Ethical standards

*Ethical approval*: All procedures performed in this study involving human participants were in accordance with the ethical standards of the institutional research committee and with the 1964 Helsinki declaration and its later amendments or comparable ethical standards.

*Informed consent*: Informed consent was obtained from all participants included in the study.

## Conflicts of interest

The authors declare no conflicts of interest.
